# Judgement analysis of case severity and future risk of disability regarding chronic low back pain by general practitioners in Ireland

**DOI:** 10.1371/journal.pone.0194387

**Published:** 2018-03-26

**Authors:** Christopher P. Dwyer, Pádraig MacNeela, Hannah Durand, Andrea Gibbons, Bronagh Reynolds, Edel Doherty, Sinéad Conneely, Brian W. Slattery, Andrew W. Murphy, Brian E. McGuire

**Affiliations:** 1 Centre for Pain Research, National University of Ireland, Galway, Ireland; 2 School of Psychology, National University of Ireland, Galway, Ireland; 3 Health Psychology Research Unit, Royal Holloway, University of London, London, United Kingdom; 4 Discipline of Economics, J E Cairnes School of Business and Economics, National University of Ireland, Galway, Ireland; 5 Discipline of General Practice, School of Medicine, National University of Ireland, Galway, Ireland; Southeast University Zhongda Hospital, CHINA

## Abstract

Chronic low back pain is a major healthcare burden that has wide ranging effects on the individual, their family, society and the workplace. However, appropriate management and treatment is often difficult, as a majority of cases are non-specific in terms of underlying pathology. As a result, there are extensive differences in both individual patient preferences for treatment and treatment decisions amongst general practitioners. The current study examined the clinical judgements of GPs in Ireland, regarding fictional patients’ case severity and future risk of disability, through judgement analysis. Judgement analysis (JA) is an idiographic regression modelling technique that has been utilised in extant healthcare research for the purpose of allocating weighting to judgement criteria, or cues, observed by professionals in their clinical decision-making. The primary aim of the study was to model two critical information utilisation tasks performed by GPs with regard to CLBP–in combining information cues to form a judgement about current case severity and a judgement about the same patient’s risk of future disability. It was hypothesised that the judgement weighting would differ across the two judgements and that judgements regarding future risk of disability would be less consistent among GPs than judgements about case severity. Results from the regression-based judgement analysis and subsequent follow-up statistical analysis provided support for both study hypotheses. Study findings are discussed in light of theory and research on judgement, clinical decision-making and chronic low back pain.

## Introduction

Chronic low back pain (CLBP) is a major healthcare burden which impacts on the individual, the family, society and the workplace. CLBP affects some 16–20% of the population [1, 2] and people with CLBP frequently present to their GP for advice and management. However, appropriate management and treatment is often difficult, as approximately 90% of cases of lower back pain are non-specific (i.e. there is no identifiable pathophysiological cause [3]. In addition, there are extensive differences in treatments recommended by general practitioners (GPs) and in patient treatment preferences [4–6]. Furthermore, extant research indicates that both patient and contextual variables influence medical judgements regarding chronic pain [7, 8]. In the context of this high level of variability, clinical judgements about case management can also vary considerably [9, 10]. Consistent with this perspective, Chibnall and colleagues [7] recommend that research investigate the influence of these variables (e.g. contextual/patient cues and type of judgement required) in chronic pain cases.

Self-reported pain severity is a guiding factor in the treatment and management of CLBP [11]. Though pain severity is based on a number of factors, such as intensity, disability and persistence [12, 13], it is not synonymous with *case severity*. Case severity can be distinguished in the literature as encompassing a much broader picture of the patient as a whole, including not only the pain experience, but also distress, work status [14], psychosocial status [15], physical functioning [13], and sleep problems [16].

While case severity is defined as the seriousness of the *current* situation of a patient, future risk of disability is based on a more complex array of physical, psychological and social factors [17–20]. Patients who have significant levels of pain-related disability are more difficult to treat and often require extensive intervention to improve overall functioning. Assessing the risk of future disability in the early stages of the pain experience (e.g. first three months) is important for the minimization and prevention of disability and prolonged suffering [17, 21].

The current study takes a novel approach in employing a regression-based statistical analysis strategy, *judgement analysis*, to assess how GPs in Ireland use available information sources to make judgements in the context of CLBP about case severity and future risk of disability. Judgement analysis (JA) is an idiographic regression modelling technique that has been utilised in extant healthcare research [22–28] for the purpose of allocating weighting to judgement criteria, or cues, observed by professionals in their clinical decision-making [29, 30]. Consistent with social judgement theory [29], judgements are regarded as an integration of the available pieces of information presented, weighted for their importance to the form of judgement required. By presenting a set of pre-designed individual cases, an idiographic analysis of each judge’s information weighting policy can be made. Judgement policies can be assessed among judges to assess whether particular information cues are relied on by most judges, the degree to which there is idiosyncrasy in the use of information cues between judges and whether particular forms of judgement are associated with high levels of uncertainty.

It is possible to differentiate between discrete judgements within the GP’s remit for CLBP management in primary care, some of which are liable to greater uncertainty than others. Notably, in this study, case severity and the future risk of disability judgements are differentiated in a temporal sense. That is, because a case severity judgement directs the judge to integrate those information cues relevant to the patient’s situation *now*, whereas a judgement of the same patient’s future risk of disability requires consideration of how the same information cues predict the patient’s situation at a specified time point in the *future*. Arguably, it is more difficult to make a future-oriented, forecasting judgement as there is no explicit framework that could be used to model this prediction.

JA may also have a further role to play in helping clinicians to understand their case management decisions. Thus, the primary aim of the study was to model two critical information utilisation tasks performed by GPs with regard to CLBP–in combining information cues to form a judgement about current case severity and a judgement about the same patient’s risk of future disability. In this context, five cues were included based on both their relevance to the biopsychosocial model of pain (i.e. a model accounting for the biological/physiological, psychological *and* social factors that influence the experience of pain) [31] and their relevance to CLBP, according to articles, textbooks, interviews with GPs and review of patient medical records [11]. The five cues were *Mobility* (i.e. mobility of the back and spine, based on visual observation and clinical assessment), *Self-esteem* (feelings about the self, based on self-report); *Sleep* (interruption and disturbance to restful sleep, excluding early morning waking); *Motivation* (patient’s self-direction and focus on treatment goals); and *Pain right now* (back pain severity and discomfort reported in the consultation).

To reiterate, though the primary aim of the study was to model two critical information utilisation tasks performed by GPs with regard to CLBP–in combining information cues to form a judgement about current case severity and a judgement about the same patient’s risk of future disability, subsequent to this investigation, two hypotheses were developed based on the rationale that (a) as a judgement, assessment of case severity is distinct from future disability; and that (b) judging current case severity is less demanding than judging future risk of disability. It was hypothesised that the weighting of information cues would differ across the two judgements. It was also hypothesised that GPs’ judgements of future risk of disability would be less consistent than judgements of current case severity (i.e. possess less internal validity of policy–see Cook & Stewart [32]). Given the absence of gold standards or normative models with respect to the accurate/correct weighting of the information cues used [11], accuracy of the judgements was not assessed.

## Methodology

### Design

A judgement analysis design was used, in which individual judges with knowledge of an issue are presented with a number of cases or instances of a particular phenomenon to review [29, 30]. The case provides a unique set of values on the critical information cues or case factors that have been chosen by the case designers to represent the phenomenon. The judge renders a judgement of each case, which represents how the information cues for that case have been assimilated by the judge.

Judgement analysis is an idiographic strategy, in that a block-wise, multiple linear regression analysis is made of the judge’s set of responses made across the cases, using the information cues as predictor variables and the judgement as a criterion variable. This procedure results in a ‘judgement profile’ that displays the relative weight attributed to each case factor across the set of presented cases Specifically, judgement analysis (JA) focuses on the weighting of importance given by decision-makers to environmental cues, based on Brunswik’s lens model [33]. JA involves allocating relative weights (in this context, based on normalized standardized regression weights; that is, the quotient of the standardized regression weights by their sum) to selected judgement criteria, as widely used and accepted in JA research [26, 34, 35].

In the current study, judges were GPs asked to respond to a set of hypothetical cases of CLBP. Separate regression analyses were conducted for the discrete tasks requested of the GP–to rate the hypothetical patient in respect of case severity and risk of future disability. Regression coefficients arising from the regression analyses of each GP’s responses were used as indicators of the importance of each cue for the diagnostic judgements; that is, their *weighting* [36].

Follow-up statistical analyses were then carried out to evaluate the study hypotheses, which included the assessment of *R*^2^ values as a measure of the concept of cognitive control [29], used in JA as an index of the coherence of an individual judge’s judgement or information use policy. Lower *R*^2^ values are associated with both unsystematic random errors and systematic but non-modelled influences. Thus, higher *R*^2^ values indicate that the judge has more control over the judgement policy and that the information cues included in the model are more directly linked to judgement ratings.

Cluster analysis was also used in the follow-up analyses. Two two-step cluster analyses were conducted to assess the hypothesis that there would be less consistency in judgements of future risk of disability compared with judgements of current case severity. Cooksey [29] describes cluster analysis as a useful method for assessing patterns in relative weight by identifying groups of judges who share a similarity in judgement policy profiles. The two-step cluster analytic method was used with relative weights for each judgement task. This method generates clusters an automatic clustering function base on Schwarz’s Bayesian Criterion (BIC) and Log likelihood calculation of distances.

### Participants

Participants were general practitioners (GPs). A list of 40 GPs was compiled from the Irish GP Training Scheme to reflect a representative sample (including rural and urban areas) contacted and invited to take part in the study. Participating GPs (*N* = 28; 20 males and 8 females) were aged between 33 and 68 (*M* = 46.93, *SD* = 8.84) were qualified between two and 34 years (*M* = 21.52; *SD* = 9.30); and reported seeing between two and 64 (*M* = 16.34, *SD* = 14.46) CLBP patients per month. Approximately 44% of GPs indicated that they have a special interest in musculoskeletal disorders, 42% indicated that they had an interest in orthopaedic medicine and 40% indicated a special interest in pain management.

### Materials and measures

Each GP responded to 34 hypothetical cases of CLBP, presented via a booklet that was mailed to them. Two separate judgements were made for each case (i.e., current case severity and future risk of disability). The GPs were asked to read the instructions page and familiarise themselves with a standard, demographic patient profile for ‘James’. In order to minimise variability in judgements outside the remit of the five cues, all ‘cases’ (i.e. James) were identical with respect to background information. For example, James’ profile was developed from qualitative data from previous research [11] and reflected a CLBP case of a 45-year old married man with three children, who has experienced CLBP for the past five years, in which the cause of pain is not clear and is manifested in a pattern of ‘flare ups’ in pain and discomfort. Previous diagnostic tests had shown no indications for surgery. He has experienced an acute flare up for the past three months and although still in employment, he is worried about the pain becoming worse. He has presented to the GP with the on-going flare up and requests for a step up in the pain relief medication (see Table 1).

**Table 1:** James’ case history profile.

Case History
James is 45 years of age, married with 3 children (aged 4–10), and supportive family. A skilled worker in a manufacturing plant, duties include some physical exertion and staff supervision. On night shift for past 12 months due to job re-assignment.
General Medical Services (GMS) patient. Average of 4 GP consultations per annum due to low back pain for past 5 years, no definitive cause apparent. Acute episodes have led to sick leave for up to two weeks in the past, with longer periods of manageable discomfort (e.g., for six months).
**Medical Treatment**
Medication history in past few years: anti-inflammatories (e.g., Difene 50-100mg twice a day), and non-opiate analgesics (e.g., paracetamol 100mg twice a day, Tramadol 50mg as needed).
Compliant with medications. Has attended physiotherapy several times, not consistent in exercise and mobilisation. Self-referred to chiropractor one year ago, reported some good effect.
No evidence of structural problems in x-ray 4 years ago and earlier this year. Magnetic resonance imaging (MRI) 2 years ago, some evidence of wear and tear (mild degeneration at lumbar spine L3/4, L4/L5 and L5/S1 disc levels). Last attended orthopaedic specialist 2 years ago, indicated no reason for surgery or invasive therapy; waiting for a repeat consultation with specialist.
**Psychosocial Observations**
Previously reported worry that pain levels will increase, fear of painful movement. Not happy at times with medical care. Previously an active sportsman. Social drinker only, no indication of abuse. Mood low at times but not diagnosed as clinically depressed. Would like to change job.
**Recent GP Consultation Notes**
In past 12 months, James had 6 consultations over back pain, not including today, with 2 significant flare ups during this time. Last 4 consultations:
**3 months ago**. Attended physiotherapy x 5 sessions but found it aggravated back pain. Only analgesia used is distalgesic as needed, mobilizing well, back strengthening. Discussion with physiotherapist, advised to discuss possible change of duties with employer/supervisor, will review in 3 weeks.
**4 weeks ago**. Relapse significant, x 2 days. Significant tenderness over right sacroiliac joint and sacrum, limited left flexion and no neurology, straight leg raising positive 90 degrees. Not sleeping well with the pain. Stressed about workload. For Difene 75mg twice a day and Bymadol cover and review in 1 week, medical certificate for 1 week.
**3 weeks ago**. Considerable discomfort from pain, prescribed Tramadol 50mg twice a day, Difene 75mg twice a day. Still not able for work, medical certificate for 2 weeks.
**1 week ago**. Still complaining of low back pain, constant, worse with activity, sometimes worse after sitting, recently getting spasms, uncomfortable in bed–wakes with pain, has woken in a sweat. Feels stressed, denies low mood. Medications not sufficient to cover pain, prescribed Tramadol 100mg twice a day, Difene 75mg twice a day, Diazepam 5mg twice a day for 5 days.

Following this standard description, 34 variations on James’ current state were presented (i.e. 34 different cases to review). Each case was presented on a separate page in a bar chart format that depicted five information cues on a 1–100 scale, namely mobility, self- esteem, sleeping patterns, motivation, and pain right now. For each of the factors, higher scores indicated a more severe problem state.

To reiterate, cues were included based on both their relevance to the biopsychosocial model of pain and their relevance to CLBP, according to articles, textbooks, interviews with GPs and review of patient medical records [11]. Although there is some variation regarding how many cues and cases can be used [37, 38], it has been suggested that the number of cases should be at least five times the number of cues; and that up to 60 cases and up to six cues is the most that should be used in any single study [39]. The current study utilised five cues within 34 cases. Definitions of the case cues were provided on a separate page that the GP was requested to keep alongside each case when working through the booklet. With respect to the psychosocial cues, broad variables were used (i.e. self-esteem and motivation), so as to not confuse GPs with potential for psychiatric illness as in the potential case of more specific cues (e.g. depression and anxiety).

### Procedure

The study was performed in agreement with the Declaration of Helsinki and was approved by the Galway University Hospital Research Ethics Committee and the National University of Ireland Galway Research Ethics Committee (12/05/05). To reiterate, a list of 40 GPs was compiled from the Irish GP Training Scheme to reflect a representative sample (including rural and urban areas) contacted and invited to take part in the study. GPs interested in taking part were sent out the assessment booklet by post for self-completion within a three-week period. All GPs provided written informed consent to participate in the study. Reminder letters were sent to those who did not return the booklet after three weeks. Each GP who took part (*N* = 28) was given an honorarium payment of €100.

After reading the instructions and familiarising themselves with the James’ background, GPs were asked to rate James’ case severity and future risk of disability at the bottom of each page in the booklet. Each judgement was based on a 1–6 likert scale, from ‘low level problem’ to ‘high level problem’ for case severity and future risk of disability. Notably, the five cues were presented on a 1–100 scale to provide GPs with specific information about each case; however, they were asked to make their judgements on a labelled (‘low level problem’ to ‘high level problem’) 1–6 likert scale in order to facilitate judgement-making without undue cognitive load [40] or decision fatigue [41]. The two separate judgement assessments were recorded for each case. By knowing the specific weighting of each of the five cues in each case, the researchers could determine, over multiple cases, which cues GPs appeared to rely on in order to make each of their clinical judgements.

## Results

Separate block-wise, multiple linear regression models were estimated for each GP for the two dependent variables (i.e. current case severity and future risk of disability). The *R*^2^ statistics indicated generally good model fit. The median *R*^2^ statistic of the current case severity regression models was 0.802 (25^th^, 75^th^ percentile = 0.751, 0.830). The median *R*^2^ for the models of risk of future disability judgements was 0.736 (25^th^, 75^th^ percentile = 0.660, 0.806).

Table 2 presents a descriptive overview of the two judgements with respect to the proportion of GPs who weighted each cue highest. Half of the participants gave pain right now the greatest weight when judging current case severity and 35% of GPs weighted mobility highest. There was more diversity in weightings of cues for the risk of future disability judgement. Besides motivation, which received the highest weighting by nearly 40% of the participants, a similar proportion of 14–18% weighting was distributed among the remaining four cues.

**Table 2:** Descriptive overview of judgements as to the proportion of GPs who weighted each cue highest.

	Current Case Severity	Future risk of Disability
Mobility	0.357	0.142
Self-esteem	0.071	0.179
Sleep	0.071	0.143
Motivation	0.000	0.392
Pain Right Now	0.500	0.143

This pattern is reflected in the profile of individual GP information cue weighting presented in Table 3, which contains the relative weight for each cue for each participant and the *R*^2^ for each regression model. Upon inspection of the data, it was noted that the relative weights for most of the cues were not normally distributed. A significant Kolmogorov-Smirnov statistic was obtained for the distribution of relative weight for two of the case severity cues (i.e. self-esteem and sleep) and four of the risk of future disability information cues (i.e. all bar motivation). As a result, the non-parametric sign test was used to assess for differences in the relative weights. With respect to case severity, the relative weighting of mobility (i.e. the highest weighted cue) was significantly higher than that of motivation (*z* = 4.347, *p* < .001). Pain right now was weighted significantly higher than self-esteem (*z* = 3.213, *p* = .001), sleep (*z* = 3.969, *p* < .001) and motivation (*z* = 4.347, *p* < .001). There were no other differences.

**Table 3:** Relative weight for each cue for each participant and the *R*^2^ for each regression model.

Current Case Severity						Future Risk of Disability					
Mob	SE	Slp	Mot	PRN	*R*^2^	Mob	SE	Slp	Mot	PRN	*R*^2^
37	23	5	8	26	.737	42	0	0	0	58	.541
39	4	7	1	49	.772	45	7	25	6	17	.417
19	28	13	7	34	.812	11	17	43	16	14	.791
52	27	0	15	6	.535	39	27	6	24	4	.384
0	11	56	8	25	.859	0	20	10	61	9	.875
22	1	1	1	75	.833	43	0	3	1	54	.801
38	11	13	13	25	.780	24	6	12	15	44	.849
54	3	0	2	41	.777	78	9	1	1	11	.656
24	15	21	7	32	.913	13	61	0	9	16	.745
90	0	6	2	3	.809	43	7	6	1	42	.746
71	0	1	0	27	.875	5	25	4	63	3	.560
10	19	31	6	34	.818	6	44	18	16	16	.769
48	1	4	4	43	.744	26	3	0	1	71	.690
23	66	1	7	3	.730	1	23	0	76	0	.831
1	1	0	0	98	.824	3	6	43	25	22	.721
37	0	1	0	62	.779	6	5	88	0	1	.395
24	42	0	11	22	.817	4	31	6	58	1	.831
51	24	4	8	15	.826	10	38	11	38	3	.728
17	4	31	2	46	.727	2	68	14	11	6	.674
14	20	22	10	34	.601	0	39	16	44	1	.639
33	1	4	16	46	.777	13	3	35	32	16	.594
32	7	1	3	57	.880	31	1	0	40	28	.871
46	0	3	4	47	.795	7	50	5	22	16	.723
0	13	5	22	60	.727	1	35	12	47	4	.808
6	22	33	1	38	.835	0	34	3	63	0	.880
21	18	34	13	14	.788	2	4	0	93	0	.946
48	12	4	5	30	.832	21	19	2	37	20	.748
45	4	18	2	32	.708	16	17	30	35	2	.675

Mob = Mobility; SE = Self-Esteem; Slp = Sleep; Mot = Motivation; PRN = Pain Right Now

### Hypothesis 1: Weighting of information cues will differ across the two judgements

With respect to differences in relative weighting of each cue across dependent variables, an analysis using the sign test revealed that relative weight of mobility was significantly higher in judgements of case severity than for risk of future disability (*z* = 2.694, *p* = .007). Similarly, pain right now was weighted significantly higher in judgements of case severity than in future risk of disability (*z* = 3.591, *p* < .001). The opposite pattern occurred in ratings of motivation (*z* = 3.334, *p* = .001) which was weighted significantly higher in judgements of future risk of disability than in case severity. There were no significant differences in ratings of sleep across the two judgement tasks.

### Hypothesis 2: GPs’ judgements of future risk of disability will be less consistent than judgements of current case severity

The coherence of the GPs’ regression models was assessed by mean *R*^2^ across the two judgement tasks and by conducting a two-step cluster analysis of the judges. A paired samples t-test revealed that the *R*^2^ for case severity (*M* = .78, *SD* = .08) was significantly higher than for the equivalent regression models referring to future risk of disability (*M* = .71, *SD* = .15; *t* = 2.80, *df* = 27, *p* = .009).

A two-step cluster analysis of the relative weights generated in response to the case severity judgement task yielded one cluster. The BIC value for a one cluster solution was 127.84, considerably smaller than the BIC value for a two-cluster solution (139.01). This suggests that there was no meaningful further discrimination of GPs into different judgement policy types when considering the use of the five information cues.

By comparison, two clusters of judges were identified on the basis of relative weights on the future risk of disability judgement task, with a BIC value of 127.27. The cluster quality was appraised as ‘good’, with a silhouette measure of cohesion and separation of 0.5. The appearance of two clusters of participants with respect to this judgement task and only one cluster in response to the case severity judgement task is indicative of there being less consistency in the GPs’ judgement policies for the future risk of disability judgement task. Nine GPs were in cluster 1, which had a cluster profile emphasising relative weights for mobility and pain right now. Among the GPs in this cluster, the mean levels of the centroids for these two information cues were 41.22 (*SD* = 15.86) and 36.55 (*SD* = 22.92), respectively. The mean value for the centroids for the other three cues was much lower, ranging from 5.88 to 9.88. Nineteen GPs were categorised to cluster 2, which was based on motivation (mean centroid value = 39.26, *SD* = 24.93), self-esteem (mean centroid value = 28.36, *SD* = 18.82) and sleep (mean centroid value = 17.89, *SD* = 22.00), with lower values on mobility (mean centroid value = 6.37, *SD* = 6.08) and pain right now (mean centroid value = 7.89, *SD* = 7.73).

## Discussion

The current study modelled the judgements made of two important judgement tasks relevant to the management of CLBP in general practice, namely current case severity and future risk of disability. The regression-based judgement analysis and subsequent follow-up statistical analysis provided support for both study hypotheses. Across the two judgements, there were significant differences in the relative weight attributable to four of the five information cues included in the judgement analysis. Support for hypothesis 1 showed that the GPs who took part in the study placed more emphasis on biomedical indicators when judging case severity, as reflected in higher relative weight associated with the pain right now and mobility information cues. They placed more weight on motivation and self-esteem when judging risk of future disability, suggesting a tendency toward endorsing a psychosocial model of disability. The second study hypothesis was supported as well, demonstrating that GPs’ judgements of future risk of disability were less consistent than their judgements of current case severity. The judgement analysis regression models of the future risk of disability judgements captured significantly less variance than the equivalent models of current case severity. This illustrates that GPs’ were less able to base a judgement of future disability on the five information cues of self-esteem, motivation, sleep, pain right now, and mobility than they were when judging current case severity. In addition, whereas only one cluster of judges could be identified through a cluster analysis of relative weights linked to case severity judgements, two clusters were identified among GPs when analysing relative weights for future risk of disability judgements. Notably, one group of GPs were characterised by reliance on the biomedical cues of pain right now and mobility to judge future disability risk. The other group, who comprised a majority of the sample, were characterised by their weighting of motivation and self-esteem when judging disability risk.

Though the current study revealed a number of interesting findings, it can be considered limited with respect to a relatively small sample size and should thus, be interpreted with caution. However, due to the idiographic and nomothetic nature of the statistical analyses, the large number of cases and multiple judgements required per case, the sample can reasonably be considered adequate; and moreover, the two types of judgements required by GPs in each of the presented cases was a novel strength within the current research. Another factor that should be considered is that though the cues in the current study were included based on both their relevance to the biopsychosocial model of pain and their relevance to CLBP, according to articles, textbooks, interviews with GPs and review of patient medical records [11], it must be noted that their inclusion should not imply that *other* important cues could not be considered. For example, other psychological factors important for the prognosis of CLBP could have been used. This is important to consider given that results suggest that biomedical cues held more weight in judging case severity; however, this weighting might have potentially been altered had different psychological and/or social factors been used. Thus, assessment of *only* the biopsychosocial factors used in this study could perhaps have implications for judgements in ‘real-life’ cases, when not provided on a fixed scale, but observed in an interaction with the patient. Ideally, a number of additional cues could have been added, but nevertheless, consistent with best practice of judgment analysis, the number of cases should be at least five times the number of cues; and that up to 60 cases and up to six cues is the most that should be used in any single study [39]. For purposes of minimising the effect of decision fatigue, the current study utilised 34 cases, hence five cues. The findings of the current study add to our understanding of how GPs interpret cases of CLBP by drawing attention to the context set by the judgement task. Significant differences in weightings of the same cues across judgement tasks suggest that GPs did discriminate between judgements. In using the same information differently, they were attempting to tailor the information to the demands of particular judgement tasks. Furthermore, the lesser degree of consistency applied to regression models of future risk of disability and the two different clusters of GPs identified in the analysis suggest that the future-oriented forecasting of disability was the more difficult and challenging of the two judgement tasks. With that said, lower levels of variance captured by a model could suggest unsystematic error or the presence of a different, systematic influence [29]. For example, GPs may have naïve theories of disability formation or be aware of other factors that may be predictive of disability [42, 43]. Nevertheless, the implication of these findings is clear—GPs require greater formal support in making judgements of CLBP patients, particularly, in the case judging future risk of disability. Moreover, the findings are relevant to ongoing issues raised in research literature regarding implementation of a biopsychosocial perspective in assessing and supporting patients with CLBP [4, 17, 31].

In conclusion, the current research revealed that GPs placed more emphasis on biomedical indicators when judging case severity and more weight on motivation and self-esteem when judging risk of future disability; and that GPs’ judgements of future risk of disability were less consistent than their judgements of current case severity, as evidenced by the judgment analysis regression models. These findings imply it is becoming increasingly important to identify the judgement ‘style’ of physicians’ judgement-making (e.g. case severity and/or future risk of disability), since this may well influence their approach to management of clinical cases. Knowing what information should be attended to and which pieces of information can be excluded are essential to judgements regarding CLBP and its treatment. Through the current research, judgement analysis has been identified as one such methodology. Building upon the findings of the current research, future investigation of GPs’ clinical judgement-making regarding CLBP patients should utilise judgement analysis as a potential means of assessing the *accuracy* of clinical judgements, not only the judgement cues used. Judgement analysis is a potentially useful way of providing structured feedback on clinical decisions as well as for teaching judgement-making in clinical settings and thus; it is recommended that future research further investigate the potential benefits of this methodology to facilitate GPs in their judgement of CLBP patients’ cases of severity and future risk of disability.

## Supporting information

S1 Data Minimal data set. (SAV)

S2 Data Individual participant data. (ZIP)
